# Midlife diagnosis of Refsum Disease in siblings with Retinitis Pigmentosa – the footprint is the clue: a case report

**DOI:** 10.1186/1752-1947-2-80

**Published:** 2008-03-12

**Authors:** Hari Jayaram, Susan M Downes

**Affiliations:** 1 Oxford Eye Hospital, West Wing, John Radcliffe Hospital, Headley Way, Oxford, OX3 9DU, UK

## Abstract

**Introduction:**

Refsum disease is a potentially lethal and disabling condition associated with retinitis pigmentosa in which early treatment can prevent some of the systemic manifestations.

**Case presentation:**

We present the cases of two brothers with a diagnosis of retinitis pigmentosa from childhood in whom Refsum disease was subsequently diagnosed midlife, after routine enquiry into hand and feet abnormalities. Subsequent treatment through dietary modification stabilised visual impairment and has prevented development of neurological complications to date.

**Conclusion:**

It is therefore important to consider the diagnosis of Refsum disease in any patient with autosomal recessive or simplex retinitis pigmentosa, and to enquire about the presence of "unusual" feet or hands in such patients.

## Introduction

Refsum disease is a potentially lethal and disabling condition associated with retinitis pigmentosa in which early treatment can prevent some of the systemic manifestations. We present the cases of two brothers with a diagnosis of retinitis pigmentosa from childhood in whom Refsum disease was subsequently diagnosed midlife, after routine enquiry into hand and feet abnormalities. Subsequent treatment through dietary modification stabilised visual impairment and has prevented development of neurological complications to date.

## Case presentation

**Figure 1:**
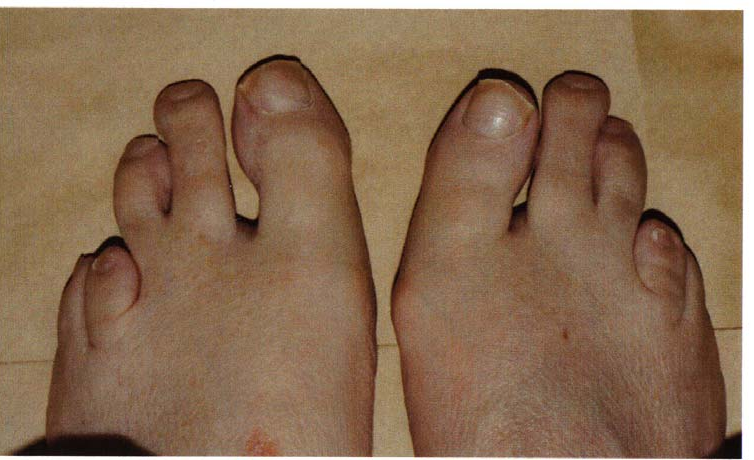
The feet of the elder sibling showed abnormal second and third toes with a shortened fourth metatarsal.

Two brothers, both Caucasian and native to South Africa, of non-consanguineous parents were referred to the retinal clinic at our hospital having recently moved to the United Kingdom. The elder brother, aged 43, was myopic and developed night blindness and peripheral visual field loss at six years of age. Following clinical examination and electrodiagnostic testing in South Africa a diagnosis of retinitis pigmentosa (RP) was made. He underwent uncomplicated cataract extraction with lens implantation in the right eye at the age of 40. He then moved to the United Kingdom and presented for review. On examination visual acuities were 6/24 OD and 6/12 OS, and due to the severity of his visual field loss he was eligible to be registered blind. On further questioning he mentioned that he had always had "unusual" feet. Examination showed abnormal 2^nd ^and 3^rd ^toes with a short 4^th ^metatarsal (Figure 1). Neurological assessment including clinical examination and electrophysiology revealed an unremarkable CNS examination with peripheral examination showing normal symmetrical reflexes and sensation with normal gait and no evidence of ataxia. A blood sample was sent for biochemical analysis, showing serum phytanic acid levels which were raised at 297 μm/L (normal range: 0–15 μm/L) with pristinate and very long chain fatty acids being within normal limits, thus confirming the diagnosis of Refsum disease (RD).

**Figure 2:**
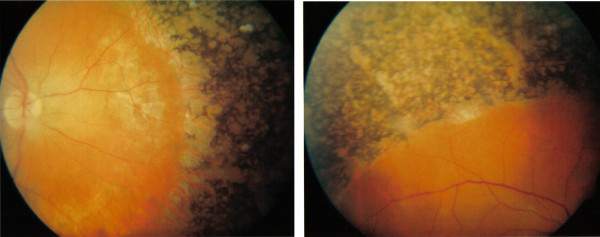
Extensive perivascular "bone spicule" pigmentation seen in both fundi of the younger sibling.

The younger brother, aged 38, had also been diagnosed with RP in South Africa at around the same time as his elder sibling. He complained of reduced taste and described a ring scotoma in his mid peripheral vision. Visual acuities were 6/6 in both eyes and perimetry revealed constricted visual fields. Examination of the ocular fundi showed extensive perivascular bone spicule intra-retinal pigmentation in the peripheral retinae (Figure 2). He also had abnormal toes with a short 4^th ^metatarsal similar to his elder brother. Electrophysiology demonstrated evidence of peripheral neuropathy and the Pennsylvanian test for olfactory sensation was reduced. Neurological assessment was otherwise unremarkable. Serum phytanic acid was found to be elevated at 265 μm/L with pristinate and very long chain fatty acids within normal limits, confirming the diagnosis of RD. There were no other family members with abnormal toes or with any other significant medical or ocular history.

Both brothers started a special RD diet and serum phytanic acid levels have been reduced significantly as a consequence. Although their visual impairment is unchanged, the younger sibling reported an improvement in his sense of taste and smell and neither sibling has shown any sign of neurological complications to date.

## Discussion

RP comprises a group of genetic conditions affecting 1 in 3000 to 4000 in the population, leading to progressive photoreceptor degeneration and visual loss [1]. RP is also seen as part of several syndromic conditions, some with severe neurological features. RD, Bassen-Kornsweig syndrome, vitamin E deficiency, and gyrate atrophy are examples of conditions within this group that are amenable to dietary modification that can influence the course of disease.

RD is an autosomal recessive disease with an incidence thought to be less than 1:250000, although the exact incidence and prevalence of the disorder in the general population is not known. Dietary phytanic acid (a branched chain fatty acid) accumulates within the body due to an abnormality in a mitochondrial enzyme phytanic acid α-hydroxylase [2]. The condition shows genetic heterogeneity with one locus on chromosome 10 [3] and a second located on chromosome 6 [4]. Phytanic acid accumulates in retinal pigment epithelium and other tissues and causes cellular death through calcium deregulation, free radical formation and apoptosis [5]. Phytanic acid is not only elevated in RD, but also in other peroxisomal disorders. However, these can be distinguished by molecular genetic analysis and clinical phenotype.

The clinical manifestations of RD affect the eyes, nervous system, bones and skin, and most patients are symptomatic before the age of twenty but may present as late as the fifth decade [6].

Bone spicule retinopathy is a universal and usually early sign in RD. Many patients have noticed night blindness prior to the onset of other symptoms and have constricted visual fields at presentation [6]. Cataract is also a frequent finding in almost 50% of all RD patients [6].

There is a symmetrical mixed motor and sensory polyneuropathy initially affecting the distal lower limbs, which is chronic and progressive in nature and usually preceded by visual symptoms. Many patients also exhibit cochlear hearing loss. Impaired sense of smell presents early in the disease and is thought to be a universal feature [7]. Cerebellar signs tend to develop later.

Bony abnormalities are seen in over a third of patients and tend to be symmetrical and bilateral in nature [8]. The short tubular bones of the hands and feet are most often affected, in particular the terminal phalanx of the thumb and the fourth metatarsal.

The skin can also be affected with rough scaly thickening seen over the extremities (ichthyosis) [9]. Cardiac abnormalities have also been reported, including cardiomyopathy and conduction disturbances, and may be responsible for causes of sudden death in RD [10]. Reports of cardiac arrhythmias, as well as neurological abnormalities indicate that Refsum patients should therefore be managed by a multidisciplinary team.

Treatment for RD is aimed at lowering the serum levels of phytanic acid. Phytanic acid comes exclusively from exogenous sources and hence dietary restriction of products rich in phytanic acid, such as dairy products and ruminant meats and fat, helps to control serum levels. Restriction of green vegetables has found to be unnecessary as chlorophyll bound phytol has poor bioavailability. Diets which are low in phytanic acid are extremely unpalatable and consequently regimens now include poultry, pork, fruit and vegetables [11].

Plasmapheresis [12] or lipopheresis [13] can be used in the event of acute arrhythmias or extreme weakness. Where dietary control has been inadequate, these treatments have been shown to help improve the clinical picture.

Maintenance of normal serum phytanic acid levels has been associated with improvement in motor nerve conduction velocities, ataxia and stabilisation of the progression of RP [14]. Retinal changes are usually irreversible and hence dietary regimens should be implemented as soon as the diagnosis is made.

## Conclusion

RD is a potentially lethal and disabling disease, which is amenable to treatment. Brief neurological screening [15] and smell testing [7] of patients with RP have been suggested as possible strategies to identify those who require formal biochemical testing in order to increase the diagnostic yield of RD. Enquiry into the presence of "unusual" feet and hands, as with the cases we have described, may also help distinguish those patients with RD from those with RP alone. However, in view of the severity of the disease, and the fact that it is treatable, phytanic acid testing should be carried out in all cases of autosomal recessive or simplex RP. Early diagnosis of the condition and initiation of an appropriate diet is vital, in order to prevent disease progression and the subsequent development of severe neurological involvement.

## Competing interests

The author(s) declare that they have no competing interests.

## Authors' contributions

SD was in charge of the care of both patients. HJ researched the literature and prepared the manuscript with critical review from SD. Both authors read and approved the final manuscript.

## Consent

Written informed consent was obtained from the patients for publication of this case report and accompanying images. A copy of the written consent is available for review by the Editor-in-Chief of this journal.
